# Effects of Pirfenidone and Collagen-Polyvinylpyrrolidone on Macroscopic and Microscopic Changes, TGF-*β*1 Expression, and Collagen Deposition in an Experimental Model of Tracheal Wound Healing

**DOI:** 10.1155/2017/6471071

**Published:** 2017-05-11

**Authors:** J. Raúl Olmos-Zuñiga, Mariana Silva-Martínez, Rogelio Jasso-Victoria, Matilde Baltazares-Lipp, Claudia Hernández-Jiménez, Ivette Buendía-Roldan, Jazmin Jasso-Arenas, Alan Martínez-Salas, Jazmin Calyeca-Gómez, Axel E. Guzmán-Cedillo, Miguel Gaxiola-Gaxiola, Laura Romero-Romero

**Affiliations:** ^1^Department of Surgical Research, Instituto Nacional de Enfermedades Respiratorias Ismael Cosio Villegas, Mexico City, Mexico; ^2^Research Direction, Instituto Nacional de Enfermedades Respiratorias Ismael Cosio Villegas, Mexico City, Mexico; ^3^Laboratory of Biochemistry, Facultad de Ciencias, UNAM, Mexico City, Mexico; ^4^Department of Morphology, Instituto Nacional de Enfermedades Respiratorias Ismael Cosio Villegas, Mexico City, Mexico; ^5^Department of Pathology, Facultad de Medicina Veterinaria y Zootecnia, UNAM, Mexico City, Mexico

## Abstract

Tracheal stenosis (TS) is a fibrosis originated by prolonged inflammation and increased transforming growth factor beta 1 (TGF-*β*1) expression and collagen deposition (CD) in the tracheal wound. Several wound-healing modulators (WHMs) have been used to modulate the tracheal healing process and prevent TS, but they have failed, justifying the need to evaluate alternative WHM. The pirfenidone (PFD) and collagen-polyvinylpyrrolidone (Collagen-PVP) decrease inflammation and fibrosis. This study assessed the effect of PFD administration and Collagen-PVP topical application on macroscopic and microscopic changes, TGF-*β*1 expression, and CD in an experimental model of tracheal wound healing. Forty Wistar rats underwent cervical tracheoplasty, were divided into 4 groups (*n* = 10), and were treated with different WHM: group I, saline solution (SS); group II, Collagen-PVP; group III, mitomycin C (MMC); and group IV, 40 mg/kg PFD. Four weeks after surgery, the macroscopic and microscopic changes, in situ TGF-*β*1 expression, and CD in posttracheoplasty scars were evaluated. The animals treated with Collagen-PVP and PFD developed less inflammation and fibrosis than animals in the other study groups (*p* < 0.05, Kruskal-Wallis) and, moreover, showed lower TGF-*β*1 expression and CD than animals in group I (*p* < 0.05, ANOVA and Tukey's test). In conclusion, PFD and Collagen-PVP decrease inflammation, fibrosis, TGF*β*-1 expression, and CD in the posttracheoplasty rats' scar.

## 1. Introduction

Fibrotic scarring is a pathological form of wound healing caused by chronic inflammation, which leads to the increased production and accumulation of extracellular matrix (ECM) components, including collagen, due to the overproduction of proinflammatory chemical mediators and profibrotic growth factors, such as transforming growth factor beta (TGF-*β*) [1–3].

The TGF-*β* family of growth factors exhibits multifunctional activities during embryonic development, the immune response, inflammation, tissue regeneration, and wound healing, wherein they regulate polymorphonuclear chemotaxis and angiogenesis and modulate the synthesis and deposition of ECM components, including fibronectin, proteoglycans, and some forms of collagen [4]. The following three isoforms of TGF-*β* have been described: TGF-*β*1, TGF-*β*2, and TGF-*β*3; the first two isoforms (TGF-*β*1 and TGF-*β*2) are profibrogenic, whereas TGF-*β*3 is anti-inflammatory and antifibrotic. The three isoforms are present in all wound-healing stages, particularly in the proliferative and remodeling phases [3, 5], and the therapeutic modulation of this factor may prevent fibrotic scarring [6].

Tracheal stenosis (TS) results from pathological wound healing and is a difficult clinical problem to solve because it may cause high morbidity and mortality. TS is defined as disproportionate fibrosis at the tracheal wound site that obstructs the lumen and prevents airflow [7].

Balloon dilatation, endoscopic laser surgery, and resection with end-to-end anastomosis of the affected tracheal segment (tracheoplasty) have been used to treat TS, but these treatments are not 100% efficacious because they also cause mucosal damage and inflammation and may promote restenosis. To decrease this risk, these procedures have been used in combination with wound-healing modulators (WHMs) in order to pharmacologically manipulate the formation of ECM components and prevent fibrosis. Specifically, mitomycin C (MMC) is the most commonly used WHM, but it has not demonstrated the desired success because it slows wound healing, predisposes patients to acute mucus accumulation, and causes defects in mucosal revascularization [8, 9]. Collagen-polyvinylpyrrolidone (Collagen-PVP) has also been used with good results but requires four weeks of intratracheal application [10], which complicates its use because patients must undergo general anesthesia. In this context, new WHMs that exhibit improved treatment efficacy are needed.

Pirfenidone (5-methyl-1-phenyl-2-[1H]-pyridone) (PFD) is an antifibrotic, anti-inflammatory, and antioxidant drug that inhibits the synthesis of collagen and other ECM components by modifying the expression of growth factors and proinflammatory cytokines (TNF-*α*, IL-1, and IL-6) as well as the expression of profibrotic growth factors (TGF-*β*), thereby modulating fibrosis. Both the experimental and clinical uses of PFD have shown good results in treating idiopathic pulmonary, renal, hepatic [11, 12], and skin [13] fibroses. However, its use in tracheal wound healing has not been studied.

The aim of the present study was to assess the effect of PFD administration and Collagen-PVP topical application on macroscopic and microscopic changes, TGF-*β*1 expression, and collagen deposition (CD) in an experimental model of tracheal wound healing.

## 2. Materials and Methods

### 2.1. Experimental Animals

Forty clinically healthy Wistar rats (strain Hsd : WI) aged 8 to 11 weeks and weighting 250 to 350 grams were used, irrespective of sex. The rats were provided by the animal house of the National Institute of Respiratory Diseases Ismael Cosio Villegas (INERICV). This protocol was approved by the INERICV bioethics committee (Permit number B23-13) and performed according to the technical specifications for the care and use of laboratory animals of the Official Mexican Standard (NOM-062-ZOO-1999) [14] and the Guide for the Care and Use of Laboratory Animals of the USA [15]. All surgeries were performed under general anesthesia, and all efforts were made to minimize suffering.

*Study Groups. *Resection and an end-to-end anastomosis (tracheoplasty) of three cervical tracheal rings was performed in all animals, which received the following treatment:

  Group I (*n* = 10): tracheoplasty with topically applied of saline solution (SS) on both ends of the resected trachea
  Group II (*n* = 10): tracheoplasty with topically applied Collagen-PVP on both ends of the resected trachea
  Group III (*n* = 10): tracheoplasty with topically applied of MMC on both ends of the resected trachea
  Group IV (*n* = 10): tracheoplasty with orally administered PFD

### 2.2. Experimental Model

#### 2.2.1. Anesthesia and Surgical Procedure

All rats were anesthetized with a 120 mg/kg intraperitoneal (IP) dose of ketamine (Anesket®, Pisa, Guadalajara, Mexico) and 12 mg/kg/IP xylazine (Rompun®, Bayer, Leverkusen, Germany).

Anesthetized rats were placed in the dorsal decubitus position, and a midline incision in the ventral cervical region was made, followed by dissection until the trachea was reached. Subsequently, the entire tracheal circumference was dissected by microsurgery (OMP 19-FC Carl Zeiss Surgical Microscope, Faserbeleuchtung, Germany) and resected from the third to the fifth tracheal ring while applying topical treatments and immediately performing an end-to-end anastomosis of the resection site with nonabsorbable 7-0 polypropylene sutures (Prolene, Ethicon, New Jersey, USA) and a simple interrupted suture pattern. Finally, conventional closure was performed. The rings resected were collected and used as control samples.

All animals subcutaneously (SC) received 4 mg/kg of flunixin meglumine (Napzin, Pisa, Mexico City, Mexico) as analgesic and intramuscularly (IM) received 5 mg/kg of enrofloxacin (Baytril, Bayer, Leverkusen, Germany) as antibiotic for five postoperative days. The animals were housed for four weeks in polysulfonate cages (26.0 cm × 47.6 cm × 20.3 cm) and provided water and feed ad libitum. The animals also received the veterinary care required for animals subjected to surgery [14, 15].

#### 2.2.2. Application of Treatment

Animals in groups I, II, and III received the treatment during surgical process on both ends of the resected trachea. The animals in group I received 0.5 mL SS, whereas those in group II received 2.5 mg Collagen-PVP (Fibroquel, Aspid S.A de C.V., Mexico City; this dose is clinically used for skin and tendon problems and has been experimentally used in chronic TS) diluted in 0.5 mL distilled water [10]. Group III received 1.2 mg/kg MMC (Mixandex, Pisa, S.A. de C.V., Guadalajara, Mexico; this dose is used for the clinical treatment of TS) [9]. In group IV, the animals orally (OR) received 2 doses of 40 mg/kg/day PFD (KitosCell, Cell Pharma, S. de R.L. de C.V., Mexico City; this dose is used to treat pulmonary fibrosis in experimental models) after surgery for 4 weeks [12, 16].

### 2.3. Evaluation

#### 2.3.1. Clinical Evaluation

The study lasted four weeks, and all animals were clinically assessed daily during the first postsurgical week, every third day in the second week, and weekly in the remaining two weeks of the study. The presence of stridor and dyspnea was primarily assessed during the study period.

#### 2.3.2. Macroscopic Evaluation

All animals were euthanized with an overdose of sodium pentobarbital (150 mg/kg/IP; Anestesal, Pfizer, S.A. de C.V., Guadalajara, Mexico) upon study completion [14, 15]. The anastomosed tracheal segment was removed, and the wound healing in the extraluminal and intraluminal tissues of the anastomosis was macroscopically evaluated. Fibrosis and edema formation, the presence of inflammation, dehiscence, and infection were assessed. The evaluation was carried out according to the presence or absence of changes and according to severity (absent, mild, moderate, and severe).

#### 2.3.3. Morphometric Evaluation

For the morphometric assessment, the anastomosed tracheal rings were placed on a millimetric scale, and photographs of the tracheal lumen were taken with the same microscope used for surgery to subsequently calculate the tracheal lumen diameter, which was compared with the diameter of the rings resected during the tracheoplasty.

At the end of this evaluation, the samples in each group were split into two portions: one for the microscopic and immunohistochemical study and the other to assess CD in the scar tissue.

#### 2.3.4. Microscopic Evaluation

For the microscopic assessment, the collected samples were fixed in 10% formaldehyde for 24 hours and subsequently embedded in paraffin. The paraffin blocks were cut into 4 *μ*m sections and stained with hematoxylin-eosin and Masson's trichrome. The presence of inflammation and fibrosis and collagen fiber organization and neovascularization were then assessed. The entire circumference of the sample was evaluated and was assessed using a semiquantitative scale, wherein a grade was assigned to each parameter assessed according to the intensity of histopathological changes (grade 1: absent 0–10%, grade 2: mild 11–25%, grade 3: moderate 26–50%, and grade 4: severe 51–100%).

#### 2.3.5. Immunohistochemical Evaluation

In situ TGF-*β*1 expression in the tracheal scar was assessed by immunohistochemistry (IHC) of the tracheal tissue sections used for microscopy with a rabbit polyclonal anti-TGF-*β*1 antibody (Abcam ab25121, USA) using the biotin-streptavidin-peroxidase system (Vectastain Universal Quick Kit, Burlingame, CA). The sections were incubated with 3,3′-diaminobenzidine (DAB, BioCare Medical, USA) and counterstained with CAT hematoxylin (BioCare Medical, USA). After completing the staining, TGF-*β*1 expression was quantified in the entire sample circumference using the program ImageJ (http://rsbweb.nih.gov/ij/), which was developed by the National Institutes of Health (NIH), and the plugin IHC Profiler [17].

#### 2.3.6. Biochemical Evaluation of Collagen Deposition

Collagen deposition in the tracheal scar was assessed using the Sircol™ soluble collagen assay, due to the fact that, after a trauma, wound healing is performed with soluble collagen [18]. Tracheal anastomosis samples were frozen after collection, lysed in 1 mL of pepsin lysis buffer (P7012 Sigma-Aldrich, USA) with a concentration of 0.001 g/1 mL of 0.5 M acetic acid (Macron Fine Chemicals™, Mexico), and incubated for 48 hours at 4°C. The total protein concentration in the supernatant was measured using the Lowry method. The quantification of soluble collagen was performed using the Sircol stain reagent which contains Syrian red and the other components of the Sircol (Kit Product: S1000, Biocolor, UK) following the manufacturer's instructions. After completion of the assay, samples were read at 555 nm using an Epoch spectrophotometer (BioTek Instruments, Winooski, USA) to quantify CD.

### 2.4. Statistical Analysis

The clinical, macroscopic, and histological data were statistically analyzed using the Kruskal-Wallis test, whereas immunohistochemical and biochemical data were subjected to analysis of variance (ANOVA) and Tukey's test (values are expressed as the mean ± standard deviation); *p* values < 0.05 were considered significant.

## 3. Results

### 3.1. Clinical Findings

All animals survived the surgical procedure. Clinically, stridor was observed during the first postsurgical week in four rats from group I, two rats from group II, and four rats from groups III and IV. This symptom disappeared in the first days of the second postsurgical week in groups I, II, and IV, while, in the rats of group III, the stridor persisted until the third week and two of these animals were euthanized because they showed signs of respiratory insufficiency.

### 3.2. Macroscopic Findings

At the end of study, all tracheal anastomoses were healed without dehiscence or infection. All (100%) animals in group I and 8 (80%) in group III developed moderate inflammation and loose fibrous tissue in the outer portion of the trachea as well as in the mucosa. The other 2 (20%) animals of group III which failed to complete the study period showed a 50% decrease in the tracheal lumen by the development of severe inflammation, as well as firm and abundant fibrous tissue in the mucosa. Moreover, only in group III, 7 (70%) rats showed mild edema (*p* = 0.001, Kruskal-Wallis). In contrast, all animals in groups II and IV showed mild inflammation and loose fibrous tissue (*p* = 0.001, Kruskal-Wallis) (Table 1).

### 3.3. Morphometric Findings

**Figure 1:**
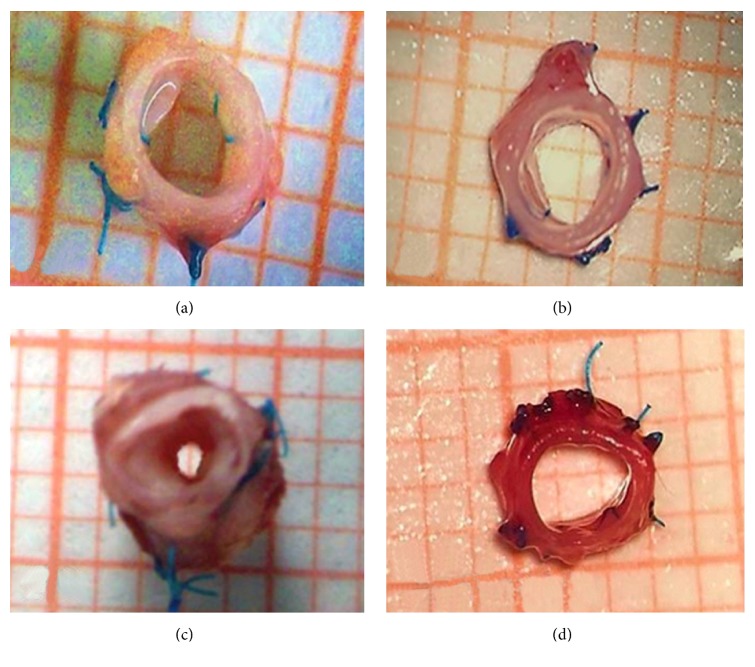
Tracheal wound healing and lumen diameter at the end of the study. (a) Normal wound healing and minimal tracheal lumen decrease in an animal from the SS group. Normal wound healing without stenosis in the group treated with Collagen-PVP (b) and PFD (d). (c) Fibrotic scarring and decreased tracheal lumen in a euthanized animal from group III.

The postsurgical diameters of the tracheal lumen in groups II (Collagen-PVP; Figure 1(b)) and IV (PFD; Figure 1(d)) (6.28 ± 0.21 mm and 6.11 ± 0.26 mm, resp.) were similar to the presurgical diameters (6.62 ± 0.06 mm). Conversely, the diameter of the tracheal lumen significantly decreased in animals from groups I (SS; Figure 1(a)) and III (MMC; Figure 1(c)) (5.57 ± 0.47 mm and 5.75 ± 0.30 mm, resp.) compared with that of control tracheal rings (6.62 ± 0.06 mm; *p* < 0.01, ANOVA and Tukey's test).

### 3.4. Microscopic Findings

**Figure 2:**
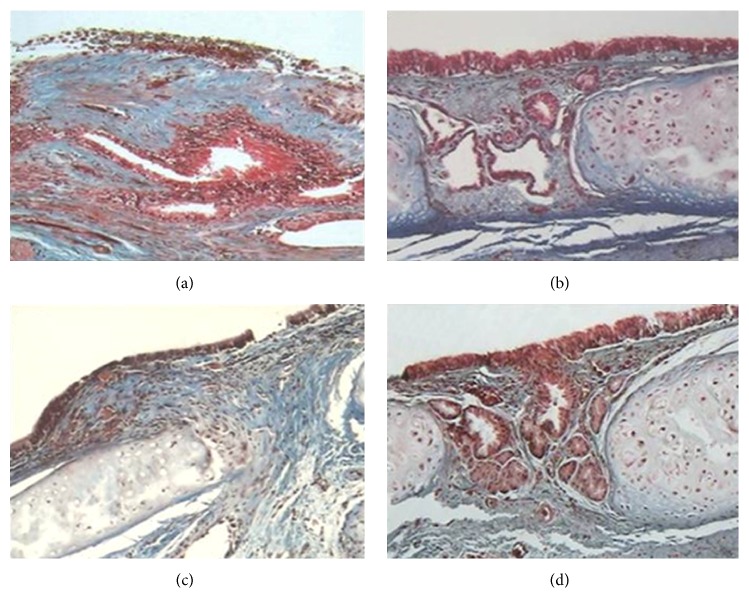
Micrograph of tracheal wound healing stained with Masson's trichrome (40x magnification). (a) Moderate inflammation and moderate amount of disorganized, thick collagen fibers in group I. (b) Mild inflammation and minimal number of organized, thick collagen fibers in the Collagen-PVP group. (c) Moderate inflammation and fibrosis with a moderate amount of disorganized, thin collagen fibers in animals from group III. (d) Tracheal wound free of inflammation and few organized, thin collagen fibers in the group treated with PFD.

All control tracheal rings were normal in appearance, whereas a partial loss of epithelium was observed in all groups at the end of the study. Furthermore, all animals (100%) from group I and one (10%) animal from group IV developed epithelial hyperplasia. Regarding the degree of inflammation, 6 (60%) and 4 (40%) of the animals in group I developed moderate and severe inflammation, respectively (Figure 2(a)). In group II, 7 (70%) of the animals treated with Collagen-PVP exhibited mild inflammation, and the remaining 3 (30%) show evidence of moderate inflammation (Figure 2(b)). In the group treated with MMC (group III), 8 (80%) and 2 (20%) of the animals developed moderate and severe inflammation, respectively (Figure 2(c)). In group IV, all (100%) rats showed mild inflammation (Figure 2(d)). Microscopic inflammation was significantly lower in the group treated with PFD than groups I and III (*p* = 0.0001, Kruskal-Wallis). Conversely, lymphocytes were the main inflammatory cells observed in all groups. Although lymphocyte expression was higher in groups I and III, significant differences in this parameter were only observed between groups I and IV (*p* = 0.0001, Kruskal-Wallis). In addition, all groups showed macrophage infiltration; however, the expression was moderate in groups I and III, as well as mild in groups II and IV (*p* > 0.05, Kruskal-Wallis). None of the animals presented neutrophilic infiltration. Regarding the degree of fibrosis, all (100%) animals of group I and 8 (80%) of group III developed moderate fibrosis (Figures 2(a) and 2(c)). The other 2 (20%) animals of group III presented severe fibrosis. Group II (Figure 2(b)) and group IV (Figure 2(d)) showed mild fibrosis (*p* = 0.001, Kruskal-Wallis). Animals from group I and III showed fibrosis with disorganized and thick collagen fibers (*p* = 0.001, Kruskal-Wallis). Group II developed collagen fibers which were organized and thick, while group IV showed organized and thin collagen fibers. The presence of newly formed blood vessels was observed in all animals of groups I (mild), II, and IV (moderate). New blood vessel formation was mild in 50% of the animals from group III and absent in the remaining 50%; these findings significantly differed from those in the other study groups (*p* = 0.005, Kruskal-Wallis).

### 3.5. Immunohistochemical Findings

**Figure 3:**
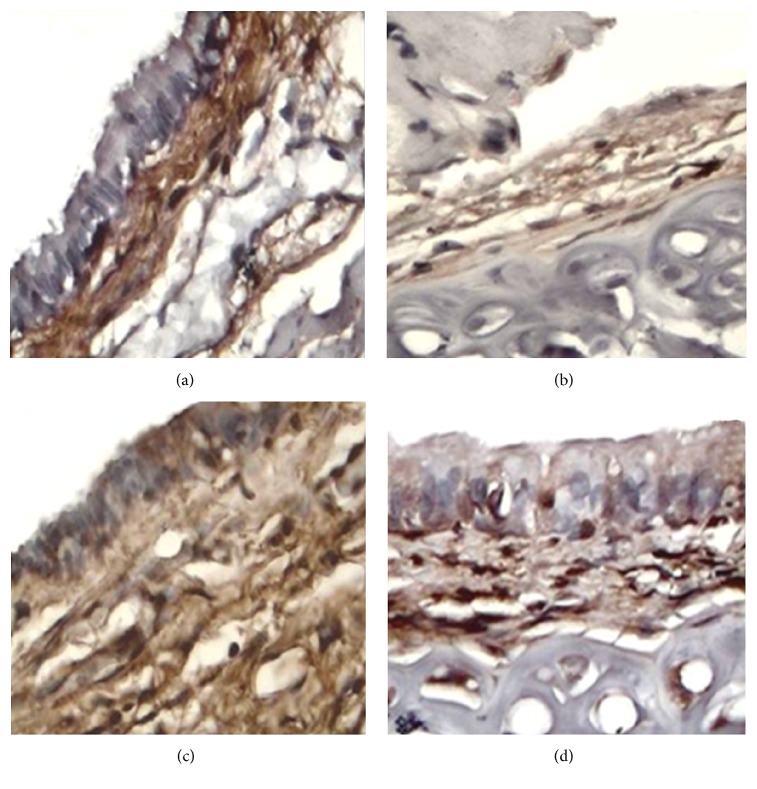
Photomicrographs of immunohistochemical detection of TGF-*β*1 in tracheal scar (40x), showing strong TGF-*β*1 expression (dark brown immunostaining) in the SS (a) and MMC (c) groups, in contrast to weak TGF-*β*1 expression (light brown immunostaining) in animals treated with Collagen-PVP (b) and PFD (d).

TGF-*β*1 was expressed (relative expression based on pixel analysis) in all groups, but its expression was lower in groups II (774245 ± 41921; Figure 3(b)) and IV (635009 ± 125667; Figure 3(d)) than in groups I (1189935 ± 91296; Figure 3(a)) and III (858936 ± 30201; Figure 3(c)). However, this difference was only significant between group I and groups II and IV (*p* < 0.05, ANOVA and Tukey's test).

### 3.6. Biochemical Findings

**Figure 4:**
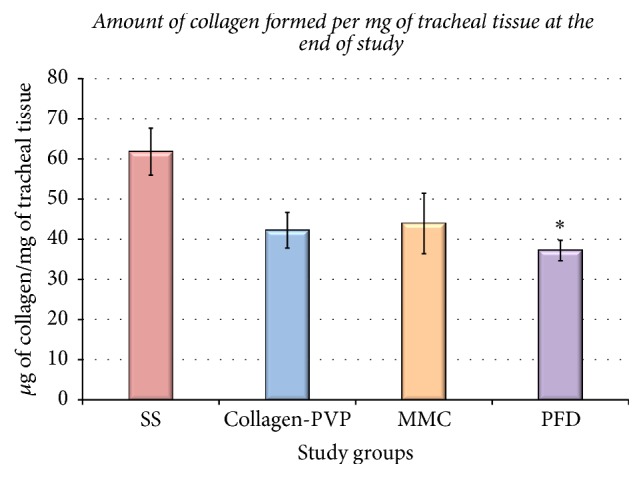
Amount of collagen formed per mg of tracheal tissue in each group, 28 days after tracheal surgery, and a comparison among groups. Each bar represents the mean ± EE of the collagen concentration in the tracheal tissue for each study group; ^*∗*^*p* < 0.05 (ANOVA and Tukey's test) showing the reduced collagen depositions observed in the PFD group versus the SS group. $\overset{-}{X}\pm SE$. ^*∗*^*p* < 0.05 ANOVA, Tukey's test, PFD versus SS.

The assessment of the amount of collagen formed after tracheoplasty per milligram of tissue showed that collagen was deposited in all animals but that collagen production was lower in the WHM-treated groups than in group I. However, this difference was only significant between group IV (PFD) and group I (*p* < 0.003, ANOVA and Tukey's test; Figure 4).

## 4. Discussion

Tracheal stenosis is a pathological scar in which the inflammatory phase of cicatrization is prolonged and TGF-*β*1 production and collagen synthesis and deposition in the ECM are increased [4, 7], which causes fibrosis at the lesion site [5, 6]. TSs are treated with endoscopic and surgical procedures combined with topical WHM application (e.g., MMC) to pharmacologically manipulate ECM formation and prevent tracheal restenosis. However, an optimal WHM that can prevent inflammation and does not require multiple endoscopic applications has not yet been identified [8–10]. Therefore, we herein assessed the effects of PFD on tracheal wound healing because this drug has shown antifibrotic, anti-inflammatory, and antioxidant properties in several tissues. Specifically, it inhibits TGF-*β* expression and collagen synthesis to modulate fibrosis [11–13].

The aim of the present study was to assess the effect of PFD administration and topical Collagen-PVP application on macroscopic and microscopic changes, TGF-*β*1 expression, and CD in an experimental model of tracheal healing.

Stridor was most likely observed in all groups because the surgical wound caused inflammation and increased the thickness of the tracheal mucosa, which resulted in this clinical sign upon airflow [3]. However, stridor disappeared during the second postsurgical week in animals from group II because of the anti-inflammatory effect of Collagen-PVP, which agrees with the findings of other studies that investigated the effect of this compound on tracheal wound healing [10]. Moreover, the effect of PFD, which was administered to group IV, on tracheal wound healing, has not yet been reported. However, PFD presumably exhibits anti-inflammatory properties, as previously observed by several studies in which acute pulmonary inflammation was experimentally treated. Specifically, these studies reported that this drug inhibits proinflammatory cell recruitment both in allergic and in nonallergic processes [11, 19]. Similarly, another study of a murine model of bleomycin-induced pulmonary fibrosis reported that treatment with this compound decreases the presence of edema caused by the lesion after 10 days, which most likely explains the decreased inflammation of the tracheal mucosa and stridor [16]. Conversely, in group III, stridor persisted for three weeks because MMC has no anti-inflammatory effect and even causes wound site irritation, fibrinoid detritus accumulation, and tissue granulation after two months of application, as reported by Roh et al. [8] and Iñiguez-Cuadra et al. [20], who clinically and experimentally studied the effects of MMC on the airway.

The tracheal edema observed after MMC application has not been fully elucidated, although the antiproliferative effect of the drug presumably delays wound healing and reepithelialization, changes endothelial morphology, and increases apoptosis and vacuole and vesicle formation, which favor edema formation, as observed by Chang [21]. Specifically, they reported the presence of edema during the early postoperative period after administering this drug in corneal studies.

The tracheal lumen decreases observed in the groups treated with SS and MMC were most likely greater because neither compound exhibits anti-inflammatory properties and both compounds favor the accumulation of immature scar tissue at the anastomotic site, which reduces the lumen of the organ. This finding agrees with those of other studies that investigated the complications caused by topical MMC application in tracheal lesions [8, 20]. Conversely, the low levels of inflammation and fibrosis in the groups treated with Collagen-PVP and PFD may have been due to the anti-inflammatory and fibrinolytic effects of both drugs [1, 2, 11, 22], which prevent the accumulation of granulation tissue at the tracheal lesion site. These findings corroborate the results from other studies using Collagen-PVP and MMC as tracheal wound-healing modulators in dogs [10, 23] and the findings by Zhou et al., who observed that animals treated with PFD developed less tracheal fibrosis in a murine model of heterotopic tracheal transplantation [24].

Animals in groups I and III developed more severe histological inflammation because the treatments applied in these groups do not exhibit anti-inflammatory activity, and our findings corroborate other studies of the use of MMC for the treatment of TS that reported that this drug causes lesion site irritation up to two months after its application, which favors inflammation [8, 20]. Conversely, animals treated with Collagen-PVP developed mild-to-moderate inflammation because this drug exhibits anti-inflammatory properties over short and long periods of administration, as previously observed in clinical and experimental studies of patients with rheumatoid arthritis, hypertrophic skin scars, scleroderma, bedsores, and posttracheoplasty scars [10, 25, 26]. The animals treated with PFD showed no-to-mild inflammation because its oxidative stress-preventive properties inhibited the recruitment of proinflammatory cells [11], as observed in the present study and reported by studies of the effects of PFD on experimental models of pulmonary fibrosis [16, 27]. However, our findings disagree with those described by Takakuta et al. [28], who studied the effects of this drug on renal protection and observed that PFD prevents fibrosis but not the presence of inflammatory infiltrate in the cortical interstitium.

Lymphocytes were detected in all groups because they migrate within the tissue during the wound-healing process to synthesize lymphokines and growth factors that stimulate fibroblasts to produce collagen [29]. Group II exhibited less lymphocytic infiltrate possibly because the compound reduces chemotaxis and lymphocyte stimulation in chronic inflammatory processes [6], which corroborates the findings of tracheal surgery studies [10, 23] and studies performed in guinea pigs with asthma treated with Collagen-PVP, which showed low numbers of lymphocytes during tissue remodeling [25]. Our findings also corroborate those by Furuzawa-Carballeda et al. [26], who assessed the safety of Collagen-PVP in vitro and in vivo and noted that this biopharmaceutical does not stimulate lymphocyte proliferation. According to Shetlar et al. [13], who studied the effect of PFD on keloid scar growth, treatment with PFD may have resulted in fewer lymphocytes.

In all cases, we observed macrophages because those cells exist in different phenotypes within the healing wound, and the influence of these cells on wound healing is through the generation of growth factors that promote cell proliferation and protein synthesis, as well as by the production of proteases and their inhibitors that influence ECM content and remodeling [30].

Neither of the animals showed neutrophil infiltration because the histological evaluation was carried out at the end of study, and these inflammatory cells usually appear during the first 24 to 48 hours following the production of the wound to remove foreign material, bacteria, and nonfunctional host cells and damaged matrix components that may be present in the wound site [3].

The decreased fibrosis and improved histological organization of collagen fibers observed in groups treated with Collagen-PVP and PFD confirm that the proinflammatory and profibrogenic cytokine-inhibiting properties of these drugs modulate fibrosis and promote the formation of collagen fibers that are organized as observed in normal tissues [1, 13, 16, 23]. These results corroborate the findings of several studies that topically applied Collagen-PVP and concluded that this drug enhances collagen production and organized CD in the ECM, as previously shown in the skin [1, 10, 31]. Similarly, our results corroborate the findings of other studies that reported decreased fibrosis and improved collagen organization after treating pulmonary [16, 27], tracheal [24], hepatic, and renal [28] fibrotic lesions and glaucoma [32] with PFD. Moderate-to-severe fibrosis with disorganized collagen fibers at the wound site was observed in MMC-treated animals because the application of this drug causes irritation and prolonged inflammation at the wound site, which favors fibrosis in the setting of TS [9, 33].

Conversely, group III likely exhibited few blood vessels because MMC blocks vascular endothelial growth factor (VEGF) expression, as described by Su et al. who studied the effect of this compound on wound healing after a laminectomy [34].

All drugs decreased the in situ expression of TGF-*β*1. Specifically, the results from group II corroborate the findings of Krötzsch et al. [1] and Furuzawa-Carballeda et al. [31], who studied the in situ expression of proinflammatory and profibrogenic cytokines in biopsies of hypertrophic scars and in skin lesions caused by scleroderma treated with Collagen-PVP and observed that TGF-*β*1 expression decreases after treatment. Furthermore, Krötzsch et al. [1] treated normal and hypertrophic cultured skin fibroblasts with Collagen-PVP and found that TGF-*β*1 expression in treated scars was lower than that in untreated normal skin samples and hypertrophic scars. Moreover, few studies assessing TGF-*β*1 expression after application of MMC, which was administered to group III, have been published. However, our findings corroborate the results from an in vitro study that reported that the application of this compound to human skin fibroblast cultures for five minutes inhibits TGF-*β*1 mRNA expression at day seven of culture. The authors of this study attributed this effect to the inhibition of fibroblast proliferation and induction of fibroblast apoptosis by the drug and concluded that this occurred because the drug inhibits fibroblast proliferation and induces fibroblast apoptosis, decreasing TGF-*β*1 and collagen expression [35]. Decreased TGF-*β*1 expression in the group treated with PFD confirms the ability of this substance to decrease the expression of this profibrogenic cytokine and transcriptionally and translationally promote the homeostatic regulation of collagen synthesis and degradation in the ECM [11, 32], as reported in experimental pulmonary [16, 27] and tracheal [24] studies.

The biochemical assessment showed that all WHMs used in this study decrease CD. Specifically, Collagen-PVP decreases the expression of TGF-*β*1 and other proinflammatory cytokines [1, 31], which corroborates the findings of other studies that used this drug alone or in combination with hyaluronic acid to improve tracheal wound healing and biochemically observed decreases in the amount of collagen formed per gram of tracheal tissue after treatment; however, Collagen-PVP was endoscopically applied four times in the aforementioned studies, whereas Collagen-PVP was only applied during surgery in the present study. This difference is important to clinical practice because the latter drug allows patients to avoid repetitive invasive procedures for treatment application [10, 23]. In group III, MMC decreased CD by blocking the proliferation and replication of fibroblasts and stimulating their apoptosis, thereby decreasing the expression levels of procollagen I and procollagen III [23, 35]. The significant difference in CD between groups IV and I may have been due to the PFD-mediated decrease in inflammation, inhibition of TGF-*β*1 expression and other profibrogenic factors, and activation of MMP synthesis, which allows this drug to modulate the fibrogenic pathway [11]. However, studies examining CD in tracheal scar tissue after treatment with PFD have not been published. Nevertheless, the present findings corroborate studies performed in models of bleomycin-induced pulmonary fibrosis [16, 27] and a model of liver cirrhosis caused by carbon tetrachloride [22]. Specifically, these studies quantified CD in the scar tissue and noted that it significantly decreases after treatment with this compound. Notably, the present findings indicate that collagen formation per mg of tracheal tissue is decreased more by PFD than by Collagen-PVP, but PFD must be applied for 28 consecutive days.

## 5. Conclusion

The present findings indicate that both the oral administration of PFD for four weeks and topical application of one dose of Collagen-PVP prevent TS by decreasing inflammation, fibrosis, TGF-*β*1 expression, and CD in the posttracheoplasty wound in an experimental model of tracheal healing. However, further experimental studies assessing the effect of these pharmaceutical drugs in animals with TS must be conducted.

## Figures and Tables

**Table 1:** Amount of animals with macroscopic changes in each study group.

Amount of animals with macroscopic findings at the end of study				
	Group I SS	Group II Collagen-PVP	Group III MMC	Group IV PFD
Wound dehiscence or infection	0	0	0	0
Mild inflammation	0	10^*∗*^	0	10^*∗*^
Moderate inflammation	10	0	8	0
Severe inflammation	0	0	2	0
Mild fibrosis	0	10^*∗*^	0	10^*∗*^
Moderate fibrosis	10	0	8	0
Severe fibrosis	0	0	2	0
Mild edema	0	0	7^*∗*^	0
Severe decrease of tracheal lumen	0	0	2	0

^*∗*^(*p* = 0.001, Kruskal-Wallis).
